# Angiotensin II and Atherosclerosis: A New Cardiovascular Risk Factor Beyond Hypertension

**DOI:** 10.3390/ijms26157527

**Published:** 2025-08-04

**Authors:** Nicola Morat, Giovanni Civieri, Matteo Spezia, Mirko Menegolo, Giacomo Bernava, Sabino Iliceto, Laura Iop, Francesco Tona

**Affiliations:** 1 Cardiology Division, Department of Cardiac, Thoracic, Vascular Sciences and Public Health, University of Padua, 35128 Padua, Italy; 2 Vascular Surgery Division, Department of Cardiac, Thoracic, Vascular Sciences and Public Health, University of Padua, 35128 Padua, Italy; 3 Translational Biomedicine Group, Department of Cardiac, Thoracic, Vascular Sciences and Public Health, University of Padua, 35128 Padua, Italy; 4 School of Health, LUM-Libera Università Mediterranea “Giuseppe Degennaro”, 70010 Bari, Italy; 5 Heart Center, Mater Dei Hospital, 70010 Bari, Italy

**Keywords:** angiotensin, atherosclerosis, inflammation, plaque instability, RAAS, hypertension, risk factor

## Abstract

The pivotal role of angiotensin II (AngII) in cardiovascular disease has been firmly established, as evidenced by a robust body of literature and the broad clinical application of AngII-inhibiting therapies. AngII type 1 receptor is the primary mediator of AngII action, and its activation initiates a multitude of cellular responses that contribute to the development of hypertension, structural changes in the heart and vasculature, and damage to target organs. This review examines AngII from a different perspective, exploring the link between the renin–angiotensin–aldosterone system and cardiovascular risk beyond hypertension, with particular emphasis on atherosclerosis development and progression.

## 1. Introduction

The renin–angiotensin–aldosterone system (RAAS) is a well-known actor in the pathophysiology of cardiovascular disease, mostly known for its regulation of blood pressure and fluid homeostasis. However, the RAAS is crucial to the pathogenesis of cardiovascular disease through systemic mechanisms such as vascular inflammation, endothelial dysfunction, reactive oxygen species production, and fibrosis [1]. Its primary effector hormone, angiotensin II (AngII), exerts its effects mainly through activation of the AngII receptor type 1 (AT1R) and is implicated in the pathogenesis of a diverse list of medical conditions, ranging from hypertension to chronic kidney diseases and from heart failure to atrial fibrillation [1]. A large amount of evidence suggests that AngII may increase cardiovascular risk through a direct effect on atherosclerotic plaque progression and stability. In this review, we first provide a brief overview of the pathophysiology of the RAAS, primarily focusing on hypertension-unrelated effects. Once this mechanistic context is provided, we will summarize the evidence regarding the interplay between AngII, its inhibiting drugs, and atherosclerosis.

## 2. Renin–Angiotensin–Aldosterone System

### 2.1. Renin–Angiotensin–Aldosterone System and Production of Angiotensin II

A detailed description of the RAAS is beyond the scope of this paper, and RAAS physiology has been thoroughly reviewed previously [2,3,4]. Herein, we report only the principal steps that lead to AngII production.

The generation of AngII is a tightly regulated enzymatic cascade initiated by renin, a circulating aspartyl protease mainly released by the granulated renin-producing cells of the afferent arterioles of the kidney. Renin cleaves angiotensinogen produced by the liver to form angiotensin I (AngI). Angiotensin-converting enzyme (ACE), mainly produced by the endothelium of the lung, removes two amino acids from AngI, converting it to AngII, the key bioactive component of the RAAS. AngII finally stimulates aldosterone synthesis in the adrenal gland by enhancing the activity of the steroidogenic acute regulatory protein and aldosterone synthase [3].

AngII exerts its diverse effects [5] not only through aldosterone production but also through stimulation of AT1R and angiotensin type 2 receptor (AT2R) and the modulation of the sympathetic tone [6,7] and of the vasopressin release [8,9].

### 2.2. Angiotensin II Type 1 and Type 2 Receptors

While both AT1R and AT2R are involved in the effects of AngII, the majority of AngII’s known cardiovascular effects are mediated by AT1R, a G-protein-coupled receptor (GPCR) expressed in numerous tissues [3]. The clinical relevance of AT1R is underscored by the association of single-nucleotide polymorphisms in the AT1R gene with an increased risk of cardiovascular conditions, such as hypertension [10], coronary artery disease [11], and myocardial infarction [12,13].

AT1R signaling is initiated by the phosphorylation of serine/threonine residues on its cytoplasmic tail by a G protein receptor kinase. The prominent role of AT1R in cardiovascular pathophysiology stems from its high expression in vascular smooth muscle cells (VSMCs), endothelial cells, and myocardial cells. In these tissues, AngII, acting via AT1R, promotes a range of detrimental effects, including inflammation, vasoconstriction, fibrosis, lipid oxidation, endothelial dysfunction, adhesion molecule expression, and myocyte hypertrophy [5,14].

Given this diverse array of effects, AT1R expression is tightly controlled, with chronic AngII stimulation leading to receptor downregulation [15,16,17]. AT2R, in contrast, often counteracts AT1R signaling through antiproliferative and proapoptotic actions [18]. AT2R activation of tyrosine or serine/threonine phosphatases inhibits AT1R signaling [19,20,21], and its deletion exacerbates neointimal formation and vascular inflammation [21]. Furthermore, AT2R agonists have demonstrated anti-fibrotic effects in preclinical models [22].

### 2.3. Renin–Angiotensin–Aldosterone System in Hypertension

Classically, both direct AT1R stimulation by AngII and AngII-triggered production of aldosterone lead to the development of hypertension [23].

As mentioned above, AngII directly induces vasoconstriction, which plays a predominant role in RAAS-associated hypertension. Moreover, AngII also induces salt and water retention, enhances the release of noradrenaline from vascular neurons and increases the response of vascular smooth muscle cells to noradrenaline [24].

Regarding water and electrolyte homeostasis, most AngII-mediated mechanisms involve the kidneys. Indeed, AngII-induced vasoconstriction of the afferent and efferent arterioles results in reduced glomerular filtration, and AngII stimulates sodium reabsorption, either through a direct effect on the proximal tubule or by stimulating aldosterone production in the renal gland. Beyond the kidney, AngII also acts centrally by stimulating vasopressin release and thirst sensation, ultimately leading to increased extracellular fluid volume [25].

As regards the interplay between the RAAS and catecholamines, both the sympathetic nervous system and RAAS are activated in hypertension, and there may be a synergistic effect between the two systems. Indeed, both the role of renal sympathetic nerve stimulation in renin release from the kidneys and the role of the RAAS/AngII in activating the sympathetic nervous system are well known [26,27,28,29].

## 3. The Effects of Renin–Angiotensin–Aldosterone System on Atherosclerosis

Various studies have been conducted on animal and human models confirming the involvement of AngII in endothelial dysfunction, neovascularization, cell proliferation, and proinflammatory effects, ultimately leading to atherosclerotic lesions. Even if the atherosclerotic process is significantly different between animals and humans [30], most studies are concordant in showing an association between AngII and atherosclerosis development and progression. These findings open up new exciting areas of research, possibly leading to major therapeutic implications in the future. While cardiovascular complications associated with the RAAS were classically thought to be mediated by hypertension, this new evidence could shift the focus to RAAS-induced atherosclerosis, redefining our approach to the entire RAAS. Notably, most of the evidence shows an association between AngII and features of plaque instability; however, as plaque rupture occurs sporadically in commonly used atherosclerosis models [31], a certain evidence of association between AngII and cardiovascular events is still lacking.

### 3.1. Animal Studies

#### 3.1.1. Neovascularization

Neovascularization plays a central role in atherosclerosis development [32]. Intriguingly, exogenous administration of AngII in mouse models increased plaque complexity by inducing intralesional hemorrhage and neovascularization, as evidenced by extensive areas infiltrated with erythrocytes and hemosiderin [33]. The accelerating effect of AngII on neointimal lesion formation was later confirmed in a histopathology/imaging study, where AngII administration was associated with irregularly developed intralesional neovasculature and expression of the neovascularization marker (CD31), reflected in hypertense signals in the vessel walls in the magnetic resonance images [34].

Clinically, large accumulations of erythrocytes and hemosiderin, indicating recent hemorrhage, and evidence of neovascularization have been linked to macrophage accumulation, foam cell formation [35], and plaque progression [35]. Lesions rich in thin-walled microvessels, often lacking smooth muscle support, are frequently observed in symptomatic and unstable atherosclerosis [36,37]. Taken together, these findings suggest that AngII induces neovascularization and intralesional hemorrhage, possibly representing a major determinant of plaque progression and instability.

#### 3.1.2. Fatty Streaks

Foam cell formation is a central event in the development of fatty streaks, which are the earliest detectable lesions of atherosclerosis. AngII promotes this process by stimulating both the recruitment of monocytes to the vascular wall and their transformation into lipid-laden macrophages [38]. In hypercholesterolemic mice, continuous infusion of AngII leads to marked macrophage infiltration and lipid accumulation in the aortic wall, even in the absence of further increases in plasma cholesterol or blood pressure, indicating a direct pro-atherogenic effect of AngII on vascular inflammation and lesion formation [39].

Additionally, in vitro studies have demonstrated that AngII enhances macrophage-mediated oxidation of LDL, increasing the generation of reactive oxygen species and promoting foam cell formation [40].

#### 3.1.3. Inflammation

Plaque inflammation plays a central role in plaque progression and rupture [41]. Therefore, an accurate definition of plaque inflammation is pivotal for detecting high-risk atherosclerotic plaques, and different markers have been proposed.

Pentratraxin 3 (PTX3) is a member of the pentraxin family and is found at the site of inflammation in response to primary inflammatory stimuli from various cell types, including monocytes/macrophages, endothelial cells, vascular smooth muscle cells, fibroblasts, and adipocytes [42]. PTX3 has been suggested as a marker of inflammatory activity and plaque instability. Notably, it has been demonstrated that AngII-treated animals display atherosclerotic plaques with more extensive staining for PTX3 than controls [34]. This suggests that AngII may lead to more inflamed and rupture-prone plaques.

Similarly, matrix metalloproteinase (MMP)-2 is a major proteinase found in atherosclerotic plaque lesions [43], and the presence of MMP-rich macrophages is a histological marker of expansively remodeled arteries with plaques that are vulnerable to rupture [44].

De Cunha et al. showed a two-fold increase in active MMP-2 in arteries from Ang II treated mice, mainly in lipid-laden macrophage areas. They also underscored the presence of normal proportions of foam cells/macrophages in both Ang II-treated mice and controls, suggesting the possibility that increased MMP-2 levels are due to increased macrophage/foam cell activation [33]. As a third example of the possible influence of AngII in plaque inflammation, it is known that in atherosclerotic vascular inflammation, more vascular cells, including vascular smooth muscle cells and monocytes/macrophages, undergo apoptosis. Defective efferocytosis of apoptotic cells may lead to the enlargement of necrotic areas, resulting in increased plaque instability [45]. AngII plays a crucial role in impairing efficient efferocytosis through its synergy with reactive oxygen species and the subsequent activation of the ADAM17/MerTK pathway, leading to unstable plaques [46].

Additionally, AngII induces cyclooxygenase-2 (COX-2) expression in cultured rat VSMCs. This effect is specifically mediated through AT1R, as it is completely blocked by the AT1R antagonist losartan but not by the AT2R antagonist PD123319. The induction of COX-2 is attributed not to increased transcription but to mRNA stabilization, a process regulated by the p42/44 MAPK (ERK1/2) and p38 MAPK signaling pathways. This mechanism contributes to VSMCs proliferation and vascular remodeling, which are key processes in the pathogenesis of atherosclerosis and vascular hypertrophy [47].

The effect of AngII is determined not only by the levels of AngII but also by the expression of its receptors; therefore, understanding how AngII and AT1R interact at the cellular level is critical. Yang et al. demonstrated that in hypercholesterolemic rabbits, the expression of AT1R was significantly upregulated in atherosclerotic aortic tissue, especially in the intimal and medial layers. In contrast, AT2R expression remained low and unchanged in normal and diseased tissue. This increase in AT1R expression correlates with heightened vasoconstrictive responses to AngII and norepinephrine, likely due to reduced nitric oxide (NO) availability and endothelial dysfunction [48], suggesting that increased AT1R expression also plays a pivotal role in determining the detrimental effects of AngII.

Given the above, it is clear that AngII is associated with different features of plaque inflammation, a well-known risk factor for plaque progression and instability.

### 3.2. Human Studies

Despite promising results in animals, research on the effects of the RAAS/Ang II on atherosclerosis in humans is still in its early stages and requires further investigation. Jukema et al. investigated the role of the RAAS in patients with suspected coronary artery disease and reported higher renin levels in patients with obstructive coronary disease than in patients with coronary microvascular dysfunction and in patients with normal or non-obstructive coronary disease [49], suggesting a potential direct effect of the RAAS on the progression of atherosclerotic plaques. Moreover, they found a significant correlation between renin levels and total plaque volume, which persisted after adjusting for the baseline characteristics of patients and the use of RAAS inhibitors. Notably, patients with high-risk plaques had significantly higher renin levels, further suggesting the role of renin in plaque progression and instability. This finding was consistent with the animal studies reported above; however, to our knowledge, it has not been confirmed in other human studies. Nonetheless, extensive evidence on the efficacy of RAAS inhibition in modulating atherosclerosis progression (*see below*) suggests an important association between RAAS activity and atherosclerosis also in humans.

## 4. Drugs Targeting the Renin–Angiotensin–Aldosterone System

ACE inhibitors (ACEi) and angiotensin receptor blockers (ARBs) target key molecular pathways of the RAAS, thereby modulating AngII activity. ACEi bind to and inhibit the zinc-containing active site of ACE, thereby reducing AngII synthesis. ACE also breaks down and inactivates bradykinin, a molecule with prominent inflammatory and vasodilatory effects; therefore, ACEi also simultaneously increase bradykinin levels [50]. In contrast, ARBs selectively antagonize AT1R and prevent AngII ligation without affecting bradykinin levels.

### 4.1. The Renin–Angiotensin–Aldosterone System Inhibition Beyond Hypertension

ACEi and ARBs are widely used as first-line treatments for hypertension, receiving class I recommendations from both the American Heart Association and European Society of Cardiology guidelines, and are known to be equally effective in reducing blood pressure and major adverse cardiovascular events (MACEs) [51,52].

Although hypertension is a major risk factor for cardiovascular disease, blood pressure reduction does not fully account for the beneficial impact of ACEi/ARBs on MACEs; therefore, other pathways must be considered [53]. Landmark trials, such as the Heart Outcomes Prevention Evaluation (HOPE) study, have demonstrated that ramipril (an ACEi) significantly reduces cardiovascular morbidity and mortality in high-risk patients, independent of its blood pressure-lowering effects [54,55]. Notably, the reduction in event rates, particularly for myocardial infarction, was much greater than what would have been expected from the modest decrease in blood pressure caused by ramipril.

Similarly, the Losartan Intervention For Endpoint reduction (LIFE) trial showed that losartan, an ARB, was superior to atenolol in reducing stroke risk in hypertensive patients with left ventricular hypertrophy [56]. The ASCOT-BPLA trial also notably demonstrated that a calcium channel blocker/ACEi-based regimen (amlodipine ± perindopril) was superior to a beta-blocker/thiazide-based regimen (atenolol ± bendroflumethiazide) in reducing cardiovascular events [57].

Notably, in both LIFE and ASCOT-BPLA, there was no clinically relevant differences in the achieved blood pressure between the groups, implying that the benefit of ACEi/ARBs had to be driven by other (still unmeasured) factors. The benefits of ACEi/ARBs on acute cardiovascular events reported in LIFE and ASCOT-BPLA are promising also from a different point of view: although a clear association between AngII levels and plaque rupture is missing due to intrinsic differences in atherosclerotic plaque features between animals and humans [30], the fact that reducing/blocking AngII protects humans from plaque rupture suggests a direct effect of AngII on plaque instability. Nonetheless, other possible pleiotropic effects of ACEi/ARBs should also be considered, such as antioxidant, antithrombotic, and profibrinolytic activities [58,59]. For example, losartan has been found to decrease platelet activation [60]. For ARBs, increased AT2R stimulation due to increased AngII serum levels may also play a role.

### 4.2. Renin–Angiotensin–Aldosterone System Inhibition and Atherosclerosis

Large trials have evaluated the role of ACEi/ARBs in stable coronary disease [54,61,62], and a meta-analysis of these trials highlighted the pivotal role that RAAS inhibition may play in patients with atherosclerosis [63].

These results demonstrate the need for further investigation into how these medications influence atherosclerosis progression to better identify patients who will benefit most from treatment.

In the following paragraphs, we review the evidence regarding the role of ACEi/ARBs in modulating atherosclerotic processes. The main results are listed in Table 1.

#### 4.2.1. Animal Studies

Extensive evidence from animal studies confirms the role of RAAS inhibition in reducing atherosclerotic plaque burden and limiting disease progression. Across multiple preclinical studies, interventions targeting AngII signaling, whether through pharmacologic blockade or genetic disruption, have been consistently associated with reduced lesion size, improved plaque composition, and attenuation of disease progression in atherosclerosis.

Initially, Johnstone et al. conducted an experiment on high-cholesterol-fed rabbits that were initially injured via balloon angioplasty, simulating early-stage atherosclerotic injury. The rabbits were then treated with ARB candesartan cilexetil for 12 weeks. A morphometric analysis of the thoracic aorta revealed a significant reduction in the overall plaque burden compared with that in the untreated controls. Importantly, this reduction occurred without changes in plasma lipid levels or blood pressure, indicating a direct vascular-protective effect. Histological examination further showed that candesartan-treated rabbits displayed collagen-rich plaques with decreased macrophage infiltration and reduced MMP activity, both of which are mechanisms directly linked to plaque destabilization and enlargement. As expected, treated animals had less frequent plaque disruption and thrombosis than controls [66].

Similarly, it was later demonstrated that genetic deletion of AT1R in ApoE−/− mice or pharmacological blockade of AT1R via valsartan profoundly impacted atherosclerotic plaque burden. Compared to AT1R-intact mice, AT1R-deficient or pharmacologically treated mice developed significantly smaller atherosclerotic lesions in the carotid artery, as measured by en face Oil Red O staining and the cross-sectional lesion area. In addition to limiting plaque expansion, AT1R deletion was associated with lower oxidative stress (i.e., lower NADPH oxidase activity) and reduced expression of inflammatory mediators such as MCP-1 and TNF-α (monocyte attractant protein-1 and tumor necrosis factor alpha, respectively), which are known to contribute to plaque growth and complexity [73]. Beyond inflammatory markers, an interesting result was the decrease in LDL-induced macrophage trapping in AT1R deficient mice, among whom this phenomenon was totally absent; further analysis showed that oxidized LDL-induced CD36 expression, FAK, and JNK2 phosphorylation were significantly attenuated in AT1R deficient macrophages, suggesting the inhibition of foam cell formation as well as macrophage emigration [65].

Zhang et al. evaluated the effects of sacubitril/valsartan and valsartan alone in ApoE−/− mice fed with a Western diet. After 12 weeks of treatment, mice receiving both sacubitril/valsartan and valsartan exhibited a marked reduction in the total area of atherosclerotic plaques, enhanced collagen content, and increased fibrous cap thickness compared with the untreated group. Notably, a significant difference emerged between the sacubitril/valsartan and valsartan groups, with sacubitril/valsartan demonstrating enhanced anti-inflammatory and plaque-stabilizing effects. This effect was quantified through en face analysis and histology of carotid sections, which showed smaller, less lipid-rich plaques and enhanced fibrotic content. Molecular assays confirmed significantly lower expression of pro-inflammatory cytokines, including IL-1β, IL-6, and VCAM-1, indicating that sacubitril/valsartan therapy could suppress key pathways involved in plaque expansion and vascular injury. The enhanced efficacy of sacubitril/valsartan over valsartan alone suggests that neprilysin inhibition, through the potentiation of endogenous natriuretic peptides, may amplify the anti-atherosclerotic benefits of RAAS blockade [67]. Nonetheless, the valsartan-alone group had significantly fewer inflamed (and thus more stable) plaques than the control group.

To further compare the benefits of RAAS inhibition in atherosclerosis progression, Hotchi et al. conducted an experiment using male Japanese white rabbits to model human atherosclerotic plaque progression. After inducing vascular injury via percutaneous transluminal balloon angioplasty, the rabbits were fed a high-cholesterol diet and divided into four groups: ACEi, ARBs, combination therapy group (both ACEi and ARBs), and a control group receiving a vehicle. The study found that both ACEi and ARB treatments increased the thickness of the fibrous cap, collagen content, and number of smooth muscle cells in the intima while reducing macrophage accumulation. ACEi treatment specifically reduced matrix metalloproteinase (MMP)-9 expression and gelatinolytic activity in the intima. In contrast, ARB treatment suppressed T-cell accumulation without affecting the gelatinolytic activity. Combination therapy did not exhibit additive effects beyond those observed with monotherapy. These results suggest that ACEis and ARBs have similar but not additive plaque-stabilizing effects, with each agent exhibiting specific mechanisms of action [64].

Finally, Strawn et al. demonstrated that losartan inhibited early atherogenesis in cynomolgus monkeys fed a high-cholesterol diet: treatment with losartan beginning at week 12 and continued for six weeks reduced fatty streak formation in the aorta, coronary, and carotid arteries by approximately 50% without altering blood pressure or plasma lipid levels. This beneficial effect was linked to decreased LDL susceptibility to oxidation, reduced serum MCP-1, and normalization of circulating monocyte CD11b expression, supporting anti-inflammatory and anti-oxidative mechanisms attributable directly to AT1R blockade [72].

#### 4.2.2. Human Studies

In human studies, evidence regarding the impact of RAAS inhibition on atherosclerosis has progressively shifted from theoretical plausibility to mechanistic confirmation through imaging and molecular analyses. Findings from the PARADIGM registry, a large prospective study utilizing serial coronary computed tomography angiography, demonstrated that RAAS inhibitor use (including ACEIs and ARBs) did not significantly alter overall plaque progression, quantified by the percent atheroma volume (PAV). However, when compared to matched untreated cohorts over a 3.9-year follow-up, RAAS inhibition caused a significant attenuation of non-calcified plaque progression in patients with elevated baseline PAV. This suggests a potential differential response to RAAS blockade based on the baseline atherosclerotic burden, with greater anti-atherogenic benefits in patients with high-risk phenotypes. Notably, this effect was specific to non-calcified, potentially vulnerable plaque components and was independent of other baseline therapies, including statins. These findings underscore the possibility that RAAS inhibition may preferentially stabilize lipid-rich, inflammation-prone plaques before they evolve into calcified, stable lesions [68], consistent with findings from preclinical animal studies.

Further supporting these observations, Clancy et al. investigated human carotid endarterectomy samples and demonstrated that AT1R blockade via irbesartan significantly reduced MMP-1 and MMP-8 secretion in plaque supernatants. As these MMPs are central mediators of fibrous cap degradation and are associated with macrophage activity in the shoulder regions of unstable plaques, their suppression suggests a direct role of RAAS antagonism in matrix preservation. Interestingly, ACE2 inhibition upregulated MMP expression, supporting the hypothesis that ACE2 exerts counter-regulatory and anti-inflammatory functions via the Ang-(1–7)/Mas receptor axis. These results indicate that the balance between ACE/AT1R and ACE2/Mas signaling is crucial for human plaque remodeling [69].

Further robust mechanistic insights were obtained by comparing the effects of irbesartan and chlorthalidone in patients who underwent carotid endarterectomy. Despite similar antihypertensive efficacy, only irbesartan reduced macrophage and T-cell infiltration, COX-2 and mPGES-1 expression, and MMP-2 and MMP-9 activities in the excised plaques. These changes were accompanied by a significant increase in collagen content and a reduction in gelatinolytic activity, indicating enhanced plaque stability. Thus, the anti-inflammatory and matrix-preserving effects of irbesartan were independent of blood pressure reduction and appeared to be mediated by the inhibition of the COX-2/mPGES-1/PGE2 axis, which promotes MMP synthesis and activity. Furthermore, this was not replicated with AT2R blockade, confirming the AT1R specificity of these molecular effects [43].

Similarly, the EFFERVESCENT trial assessed carotid atherosclerosis using high-resolution magnetic resonance imaging over 24 months in a double-blind placebo-controlled design. Treatment with valsartan led to significant regression in the vessel wall area and mean wall thickness at the carotid bulb as well as reductions in maximum plaque thickness, independent of blood pressure control or statin therapy. Biochemical analyses revealed improved oxidative stress parameters, such as reduced cysteine-glutathione disulfide levels, and trends toward enhanced endothelial-independent vasodilation, consistent with the amelioration of redox-sensitive signaling pathways. Importantly, these structural and biochemical effects occurred in patients without overt cardiovascular disease, suggesting that AT1R antagonism may prevent early atherosclerotic changes and stabilize preclinical lesions [70].

Carotid imaging to investigate the role of RAAS inhibition in the progression of atherosclerosis was also used in the SECURE study, a sub-analysis of the HOPE trial. In this study, patients were randomized to receive either ramipril or vitamin E (an antioxidant), and atherosclerosis progression was evaluated using B-mode ultrasonography. ACEi therapy retarded the progression of atherosclerosis (measured as the progression slope of the mean maximum carotid intimal medial thickness), whereas vitamin E had a neutral effect on atherosclerosis progression. Notably, most study patients did not have a history of hypertension or had well-controlled blood pressure, and the beneficial effect of ramipril on atherosclerosis remained statistically significant after adjusting for blood pressure changes, suggesting that the benefit was not fully explained by blood pressure lowering and may be related to a direct vascular protective effect [71].

Taken together, human studies assessing the role of RAAS inhibition in atherosclerosis are concordant in reporting a significant and robust effect of ACEi/ARBs in preventing plaque progression and stabilizing pre-existing plaques.

## 5. Future Perspectives

The above reported evidence is concordant in showing the detrimental effect of the RAAS, and specifically AngII, on atherosclerosis progression and, even more robustly, the benefit of ACEi/ARBs on plaque composition and stability. Nonetheless, these concepts have not yet been translated into everyday clinical practice, and ACEi/ARBs are mainly used as drugs for hypertension and heart failure. While we believe in the central role of AngII in the atherosclerotic process, we recognize that large studies confirming the role of ACEi/ARBs in this field are still lacking. In our opinion, computed tomography (CT, either at the carotid or coronary level) or magnetic resonance imaging (MRI, allowing for optimal characterization of peripheral atherosclerotic plaques [74]) might represent the perfect tools to be used in future studies, allowing for accurate non-invasive plaque characterization. Ideally, clinical trials should randomize patients to ACEi/ARBs and examine plaque progression using serial CT/MRI imaging. Similarly, AngII levels could be measured in patients not on ACEi/ARBs and with known atherosclerotic plaques to evaluate whether those with higher AngII levels might develop plaque progression despite conventional anti-atherosclerotic treatment (i.e., statin). This approach could help identify subgroups of individuals most likely to benefit from targeted RAAS inhibition but could also pave the way for unexplored therapeutic strategies, such as the administration of ARBs in normotensive patients with high AngII levels to prevent atherosclerosis progression. The role of newer RAAS-modulating agents, such as sacubitril-valsartan, or specific AT1R antagonists, such as sparsentan [75], also warrants further investigation. Moreover, given the increasing body of evidence showing how specific autoantibodies targeting AT1R play a relevant role in the setting of acute coronary syndromes [76,77,78,79], future research should also evaluate whether these autoantibodies have a role in atherosclerosis progression and plaque instability.

**Figure 1:**
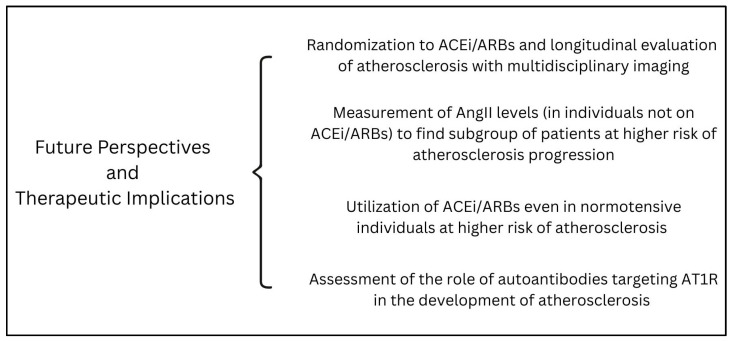
A summary of possible future perspectives and therapeutic implications of the role of angiotensin II in atherosclerosis development and progression. Abbreviations: AngII: angiotensin II; ACEi: angiotensin-converting enzyme inhibitors; ARBs: angiotensin II receptor blockers; AT1R: angiotensin II receptor type 1.

Possible future perspectives and therapeutic implications are shown in Figure 1. If the effectiveness of ACEi/ARBs in the treatment of atherosclerotic plaques is confirmed, it will provide a safe, globally approved, and inexpensive option for treating the leading cause of death worldwide.

## 6. Conclusions

The RAAS and its main effector AngII are well-known players in the pathophysiology of cardiovascular disease, and their association with hypertensive disorders has been known for decades. AngII, through activation of AT1R, also plays a pivotal role in cardiovascular disease beyond its hypertensive effects, particularly in the development and destabilization of atherosclerotic plaques.

**Figure 2:**
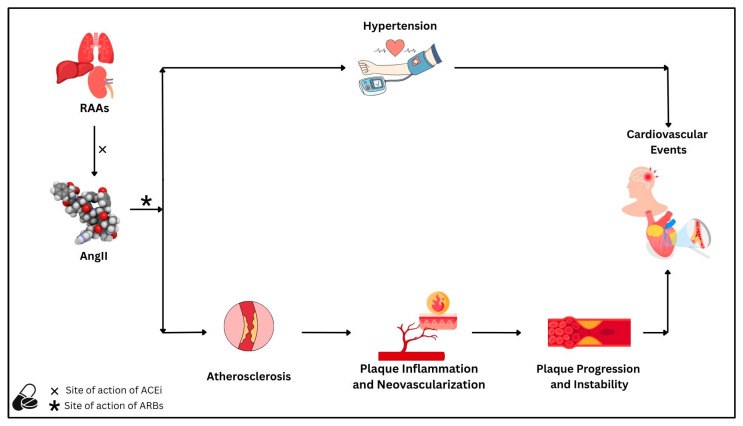
The renin–angiotensin–aldosterone system, through its main effector angiotensin II, is associated with the risk of cardiovascular disease due to (1) an increased risk of hypertension and (2) the promotion of atherosclerotic processes. Regarding atherosclerosis, angiotensin II leads to the progression of atherosclerotic plaques through mechanisms related to plaque inflammation and neovascularization. Both processes ultimately lead to plaque instability, a major driver of subsequent cardiovascular events. Angiotensin-converting enzyme inhibitors and angiotensin II receptor blockers reduce cardiovascular risk by reducing blood pressure, slowing atherosclerosis progression, and stabilizing atherosclerotic plaques. Abbreviations: AngII: angiotensin II; ACEi: angiotensin-converting enzyme inhibitors; ARBs: angiotensin II receptor blockers; RAAS: renin–angiotensin–aldosterone system.

Experimental studies in animal models have consistently shown that RAAS activation promotes vascular inflammation, neovascularization, oxidative stress, matrix degradation, and impaired efferocytosis, all of which contribute to plaque progression and vulnerability. These molecular and cellular effects are corroborated by human studies, which demonstrate that RAAS inhibition via ACEi or ARB not only reduces blood pressure but also stabilizes atherosclerotic lesions through anti-inflammatory, anti-fibrotic, and antioxidant mechanisms (Figure 2).

Overall, these findings support the concept that AngII is a critical mediator of atherosclerotic disease progression and that RAAS-targeted therapies may confer vascular protection beyond hemodynamic effects. Further studies are needed to stratify patients who may derive the greatest benefit and explore the full therapeutic potential of RAAS inhibition in atherosclerotic cardiovascular disease.

## Figures and Tables

**Table 1:** An overview of the main studies assessing the role of RAAS inhibition in atherosclerosis.

Study	Model	Endpoint	Major Findings
**Hotchi et al.** [64]	Male Japanese white rabbits fed a high-cholesterol diet after balloon injury of the carotid arteries.	Compare the efficacy and mechanism of plaque stabilization by ACEI or ARB and to determine the effects of combination therapy	ACEI or ARB increased the thickness of the fibrous cap, collagen content and the number of smooth muscle cells in the intima and reduced the accumulation macrophages, suggesting the plaque-stabilizing effect. ACEI reduced MMP-9, while ARB did not.
**Aono et al.** [65]	(ApoE)−/− and ApoE−/− AT1a−/− mice	Assess the role of AT1R in plaque rupture	Blocking AT1R may reduce atherosclerotic plaque rupture and AT1R mediated macrophage trapping, inflammation, oxidative stress, and matrix metalloproteinase activation.
**Johnstone et al.** [66]	New Zealand white rabbits fed a high-cholesterol diet after aortic balloon injury	Assess ARB effect on atherosclerosis progression	ARB attenuates the degree of atherosclerosis and reduces both plaque disruption and macrophage accumulation while increasing collagen deposition in the aorta of this animal model.
**Zhang et al.** [67]	(ApoE)−/− mice fed a high-cholesterol diet after carotid injury	Compare the effect of the sacubitril/valsartan (LCZ696) combination versus valsartan alone	Both valsartan and LCZ696 decreased plaque lipid content and cross-sectional plaque area and increased fibrous cap thickness. LCZ696 performed the best in suppressing atherosclerosis and inhibiting the level of pro-inflammatory genes.
**Williams et al.** [68]	Human without history of CAD	Asses RAAS inhibitor impact on atherosclerosis progression	RAAS inhibition caused a significant attenuation of non-calcified plaque progression in patients with elevated baseline percent atheroma volume.
**Clancy et al.** [69]	Atheroma samples obtained from patient undergoing carotid endarterectomy	Asses ATR1 and ACE1 inhibitor on the expression and activity of MMP-1, -8 and -13	AT1R blockade via irbesartan significantly reduced MMP-1 and MMP-8 secretion in plaque supernatants.
**Cipollone et al.** [43]	Atheroma samples obtained from patient undergoing carotid endarterectomy	Asses AT1R inhibitor effect on the inflammatory infiltration and expression of COX-2/mPGES-1 and MMPs	Irbesartan decreased inflammation and inhibited COX-2/mPGES-1 expression in plaque macrophages.
**Ramadan et al.** [70]	Patient with carotid intima media thickness > 0.65 mm by ultrasound	Asses AT1R inhibitor effect on carotid wall atherosclerosis	AT1R blockade was associated with regression of carotid atherosclerosis
**Lonn et al.** [71]	Patients with vascular disease or diabetes and at least one other risk factor	Compare the effect on atherosclerosis progression of ramipril versus vitamin E	Treatment with ramipril had a beneficial effect on atherosclerosis progression, while the effect of vitamin E was neutral.
**Strawn et al.** [72]	Male cynomolgus monkeys fed with a high-cholesterol diet	Assess ARBs effect on atherosclerosis progression	Treatment with losartan inhibited fatty-streak formation
